# ESG guidance and artificial intelligence support for power systems analytics in the energy industry

**DOI:** 10.1038/s41598-024-61491-8

**Published:** 2024-05-18

**Authors:** Qingjiang Li, Guilin Zou, Wenlong Zeng, Jie Gao, Feipeng He, Yujun Zhang

**Affiliations:** 1 China Southern Power Grid Co., Ltd., Guangzhou, 510000 People’s Republic of China; 2 Energy Development Research Institute, CSG., Guangzhou, 510000 People’s Republic of China; 3 Institute of Quality Development Strategy, Wuhan University, Wuhan, 430072 People’s Republic of China; 4 Yulin Power Supply Bureau, Guangxi Power Grid, Co., Ltd., CSG., Yulin, 537000 People’s Republic of China

**Keywords:** Energy science and technology, Energy infrastructure, Renewable energy

## Abstract

In order to increase the precision and effectiveness of power system analysis and fault diagnosis, this study aims to assess the power systems in the energy sector while utilizing artificial intelligence (AI) and environmental social governance (ESG). First, the ESG framework is presented in this study to fully account for the effects of the power system on the environment, society, and governance. Second, to coordinate the operation of various components and guarantee the balance and security of the power system, the CNN-BiLSTM power load demand forecasting model is built by merging convolutional neural network (CNN) and bidirectional long short-term memory (BiLSTM). Lastly, the particle swarm optimization (PSO) algorithm is used to introduce and optimize the deep belief network (DBN), and a power grid fault diagnostic model is implemented using the PSO technique and DBN. The model’s performance is assessed through experimentation. The outcomes demonstrate how the CNN-BiLSTM algorithm significantly increases forecasting accuracy while overcoming the drawback of just having one dimension of power load data. The values of 0.054, 0.076, and 0.102, respectively, are the root mean square error (RMSE), mean absolute error (MAE), and mean absolute percentage error (MAPE). Effective processing of large-scale nonlinear data is achieved in the area of power grid fault diagnosis, resulting in prediction accuracy of 96.22% and prediction time of only 129.94 s. This is clearly better than other algorithms and increases fault prediction efficiency and accuracy. Consequently, the model presented in this study not only produces impressive results in fault diagnosis and load demand forecasting, but also advances the field of power system analysis in the energy industry and offers a significant amount of support for the sustainable and intelligent growth of the energy industry.

## Introduction

Environmental Social Governance (ESG), with its emphasis on social responsibility, environmental friendliness, and good governance, has emerged as a key indicator of enterprise operation in today’s energy industry, as society’s concerns about sustainable development and environmental protection grow^1,2^. ESG covers three aspects: environment, society, and governance, and also plays a vital role in power system analysis^3^. In this context, power system analysis has become the key to ensure the success of energy industry in terms of sustainability and efficiency.

ESG guidance has a far-reaching impact on power system analysis. It urges energy companies to adopt more environmentally friendly and socially responsible practices and encourages them to integrate renewable energy into the power system. This requires in-depth analysis of the traditional power system to determine how to better integrate renewable energy, optimize energy production and distribution, and minimize the negative impact on the environment^4^. Under the guidance of ESG framework, power system analysis is not only the optimization of technology, but also the comprehensive consideration of environment, society, and governance.

In addition, the support of artificial intelligence (AI) also brings new possibilities for power system analysis. AI technology can process large-scale data, provide more accurate and real-time forecasting and optimization models, and help power systems run more efficiently^5,6^. In order to find possible areas for improving energy efficiency and to optimize the operation of the power system, which lowers energy waste and emissions, the deep learning algorithm can be used to extract features of data information from the power system in the energy industry without supervision^7–9^.

Therefore, this study aims to use ESG guidance and AI support to analyze the power system efficiently, and realize the sustainable development goal with the efficiency and reliability of the energy industry. The innovations of this study are as follows: Firstly, ESG framework is introduced into power system analysis, and combined with deep learning algorithm, power load demand forecasting and power grid fault diagnosis are improved and optimized. Second, this study proposes a CNN (Convolutional Neural Network)-BiLSTM (Bidirectional Long Short-Term Memory) power load demand forecasting model. This model is intended to facilitate the coordination of various components’ operations and guarantee the stability and security of the power system. Lastly, this study also suggests a power grid failure diagnostic model based on the particle swarm optimization (PSO) algorithm and deep belief network (DBN), taking into account the new difficulties brought about by the idea of a smart grid. This comprehensive analysis method not only helps energy companies to enhance their competitiveness, but also promotes the development of the whole industry in a more environmentally friendly, socially responsible and sustainable direction.

The overall organizational structure of this study is as follows. “Introduction” section, introduces the research background and motivation, and emphasizes the importance of power system analysis and fault diagnosis in the sustainable development of energy industry. “Recent related work” section, the recent related work, combs the related research in the field of power system analysis and fault diagnosis, highlighting the existing research results and existing research gaps. “Methods” section, the method, describes the application of ESG framework in power system analysis in detail, and designs a model based on CNN-BiLSTM and PSO-DBN in power load demand forecasting and power grid fault diagnosis. “Results” section, the result, presents the actual data and analysis results of power system analysis and fault diagnosis in the form of charts and data. “Discussion” section, the discussion, interprets and analyzes the results in detail, highlighting the significance, limitations, and future research direction of the research results. “Conclusions” section, the conclusion, summarizes the whole research and emphasizes the significance to the sustainable development of energy industry and the focus of future research.

## Recent related work

The trend in energy transformation is toward cleaner, sustainable, and environmentally friendly development due to the world economy’s rapid growth. Many scholars have studied the power system in the energy industry. Yao et al.^10^ deeply studied the renewable energy target of China in 2030, and emphasized the economic and climatic advantages of vehicles to power grid technology. Their research illustrated how this method can promote the low-carbon transformation of power system, and provided inspiration for China’s renewable energy goals. Mao et al.^11^ discussed the role of energy storage in planning low-carbon distributed power systems. Their assessment highlighted the key role of energy storage in achieving a more sustainable energy infrastructure. Huang et al.^12^ proposed a low-carbon economic scheduling and energy sharing method for multiple integrated energy systems. Their method focused on the overall perspective of the system and provided a comprehensive idea for realizing the low-carbon goal of energy distribution. Xin-gang and Ying^13^ examined China’s renewable energy industry policy and its effectiveness in supporting low-carbon energy transformation. Their work has made an in-depth evaluation of policies and measures in promoting the sustainable energy pattern. Borowski and Karlikowska^14^ solved the challenges faced by enterprises in adopting clean hydrogen in the era of low-emission and zero-emission economy. Their research emphasized the complexity and obstacles faced by integrating clean hydrogen in achieving sustainable energy goals. Wang^15^ used quantitative analysis method to analyze the data of renewable energy enterprises to determine whether there was a relationship between low-carbon transformation and ESG disclosure. The findings demonstrated a positive association between renewable energy enterprises’ ESG disclosure and their low-carbon transition. This showed that the low-carbon transformation may promote the ESG practice of renewable energy enterprises and increase the concern about environmental, social and governance issues.

Deep learning algorithms are currently being used by numerous academics to examine and research the electricity system. The use of deep learning in frequency analysis and regulation of contemporary power systems was examined by Zhang et al.^16^. They gave detailed insights into the future application of deep learning technology and talked about how it could enhance frequency analysis and power system control. Yang et al.^17^ put forward an intelligent data-driven method, which had the ability of autonomous learning and provided a new decision-making idea for the security and efficiency of power system. Hong et al.^18^ conducted power system fault event analysis based on deep learning technology. Their research used deep learning to analyze power system fault events, which provided new possibilities for improving power grid reliability and rapid fault location. Khattak et al.^19^ proposed a hybrid model based on deep learning, which was used to detect power loss using big data in power systems. Their model combined deep learning and big data technology, providing a new and efficient method for power loss detection. Ahmadian et al.^20^ proposed a method combining mixed integer linear programming with deep learning to forecast the power load demand of virtual power plants. The results showed that this method can integrate renewable energy and electric vehicles more effectively, and provide a new intelligent scheme for power system management.

To sum up, through the research and analysis of the above scholars, it is found that it highlights the diversity and complexity in the field of energy transformation and expands the understanding of how to realize a clean, sustainable and environmentally friendly power system more effectively. Deep learning, meanwhile, offers a new technical avenue for power system analysis, as demonstrated by the fault analysis of the power system by Hong et al. and the power loss detection by Khattak et al. However, in the demand of sustainable development of low carbon and environmental protection, few scholars apply ESG and AI technology to the analysis of power system at the same time. In order to encourage the growth of the global new energy field in a low-carbon and intelligent direction, this study uses the power system in the new energy field as its object, applies the ESG framework to it, and uses deep learning to analyze the power system.

## Methods

### Application of ESG framework in power system analysis

ESG framework plays a vital role in the energy industry, covering three aspects of environment, society and governance, and has become a key indicator for enterprises and industries to evaluate sustainability and social responsibility^21,22^. In the energy industry, especially in power system analysis, ESG framework plays a guiding and evaluating role, as displayed in Table 1.

**Table 1:** Application of ESG framework in power system.

Aspect	Index	Specific content	Classification	Representative meaning
Environment	Energy type	Renewable energy/traditional energy usage ratio	Less than 25%	The lower the proportion, the lower the utilization of renewable energy, while the higher the proportion, the more the enterprises rely on renewable energy, the less the dependence on traditional energy and the more environmentally friendly
			25–50%	
			50–75%	
			75% or above	
	Carbon emissions	Carbon emissions generated during power generation	Less than 1000 tons	Low carbon emissions mean that enterprises produce less greenhouse gases in the process of power generation and have less impact on the climate, while high carbon emissions mean that enterprises may have a greater negative impact on the climate
			1000–5000 tons	
			5000–10,000 tons	
			10,000 tons or above	
	Ecosystem impact	Influence of power generation on local ecosystem	No impact	No impact means that enterprise activities have no significant impact on the ecosystem, while significant impact may mean that enterprise activities have seriously damaged the ecosystem
			Slight impact	
			Medium impact	
			Significant impact	
Society	Social responsibility project	Social responsibility projects and plans implemented	Less than 5 projects	The greater the number of social responsibility projects, the more active enterprises are in fulfilling their social responsibilities and making contributions to the community
			5–10 projects	
			10–15 projects	
			15 projects or above	
	Employee welfare	The company’s welfare treatment and safety measures for employees	Less than 1000 yuan/month	The higher employee welfare means that enterprises care more about employees and provide better wages and safer working environment
			1000–3000 yuan/month	
			More than 3000 yuan/month	
Governance	Power system governance structure	Structure and decision-making mechanism of power system	Low level	The higher the level, the more transparent and independent the decision-making process of the enterprise, which reduces the possibility of power interference
			Medium level	
			High level	
	Transparency and compliance	Transparency and compliance of power system operation	Low level	The higher the level of transparency and compliance, the better the enterprise is in information disclosure and compliance with laws and regulations, and more responsible to stakeholders
			Medium level	
			High level	

In Table 1, the application of ESG framework in power system is mainly reflected in three aspects: environment, society and governance. In terms of environment, ESG framework pays attention to sustainability by evaluating the impact of power system on the environment. Through the ESG framework, energy companies can more comprehensively evaluate the environmental impact of their power production methods, thus promoting a more environmentally friendly and sustainable energy production model. In the social aspect, ESG framework also pays attention to the impact of power system on society, such as employee welfare, the impact of local communities and social responsibility. In power system analysis, social indicators may include the implementation of social responsibility projects, employee welfare and safety measures. By considering these factors, the social sustainability of power system can be evaluated and the development of social justice and responsibility can be promoted. In terms of governance, this involves the effectiveness of regulatory compliance and decision-making transparency. Through these indicators, the good governance of power system can be evaluated.

Therefore, the application of ESG framework in power system analysis not only helps to evaluate the sustainability and environmental friendliness of power system, but also promotes the development of energy industry towards more social responsibility and governance norms. In order to support the more standardized and effective operation of the power system in the energy industry, the deep learning algorithm is further introduced by this study to anticipate the power demand and fault detection in the power system.

### Application of deep learning to power load demand forecasting and analysis

This study uses a deep learning algorithm to predict power demand, coordinate the operation of various components, and guarantee the security and balance of the power system to analyze the energy industry’s power system and lessen the high fluctuation and uncertainty of the power grid load brought on by residents’ behavior.

**Figure 1:**
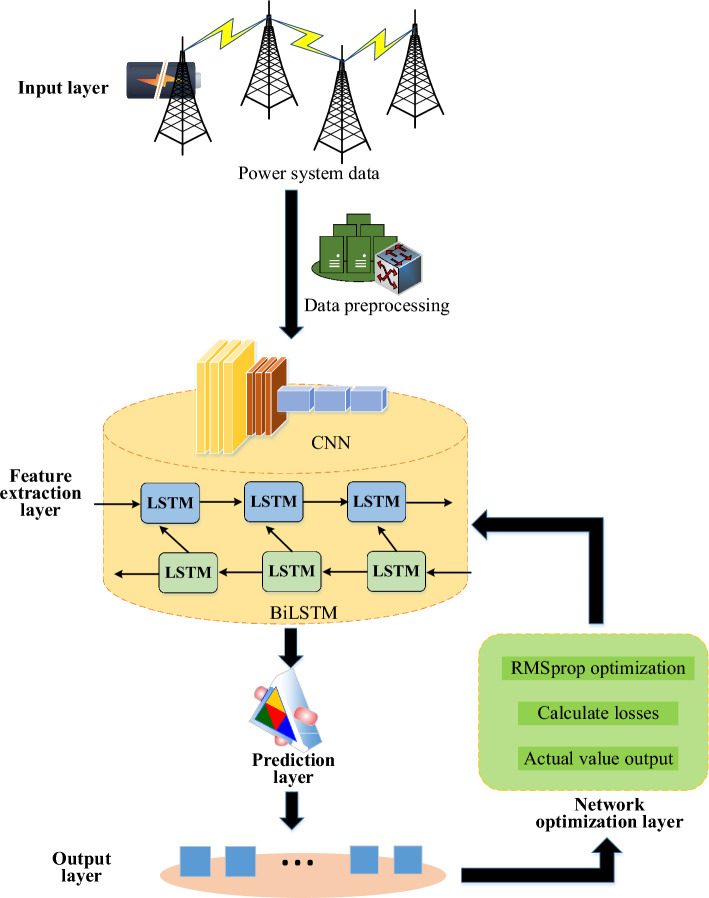
Frame diagram of power load demand forecasting model based on CNN-BiLSTM.

The one-dimensional nature of power load data significantly restricts the neural network’s capacity to extract the full range of information from power load series. Implicit qualities include the link between power load data and time series data continuity. CNN^23^ has the ability to map one-dimensional data into multi-dimensional data, completely utilize the information contained in power load series, and enhance the features of restricted data. CNN is therefore able to adequately represent the regional features of power load data. Meanwhile, power load data sequentially records power consumption based on a predetermined time interval and sampling frequency. Therefore, this study further introduces BiLSTM algorithm^24^ to extract the features of power load data. Eventually, a CNN-BiLSTM-based model structure for power load demand forecasting is built, as seen in Fig. 1, including input, feature extraction, prediction, output, and network optimization layers.

In Fig. 1, the power load demand forecasting model mainly consists of two parts: feature extraction and data forecasting. After preprocessing the data in the power system, CNN extracts the feature information sequence from the pre-processed time series. CNN module uses two convolution layers and ReLU as the activation function to make the network more trainable.

### Brief introduction and working principle of CNN-BiLSTM model

CNN-BiLSTM model combines CNN and BiLSTM to process time series data, such as the data in power load demand forecasting. Among them, CNN model consists of convolution layer, pooling layer, and activation function, which can effectively extract features from one-dimensional data. In power load demand forecasting, CNN is used to transform one-dimensional power load data into multi-dimensional data to better capture the spatial characteristics of the data. Through convolution operation and activation function, CNN can extract key features from data, such as load fluctuation and trend. In power load demand forecasting, CNN model is used for feature extraction, which transforms the original one-dimensional power load data into multi-dimensional feature sequence, so that the subsequent forecasting model can better understand and process the data.

BiLSTM model is composed of forward and backward LSTM networks, which captures the context of sequence data respectively. BiLSTM can capture the long-term dependence in sequence data to better understand the time dynamic characteristics of data. Through the forward and backward LSTM networks, BiLSTM can consider the data of the current moment and the data of the past moment at the same time to predict the future trend. In power load demand forecasting, BiLSTM model is used for sequence forecasting, receiving the feature sequence extracted from CNN model, and forecasting the future power load demand by learning the time dependence of sequence data.

CNN-BiLSTM model combines the feature extraction ability of CNN with the sequence modeling ability of BiLSTM, and makes full use of the spatial and temporal dynamic characteristics in time series data. Through this integration, the model can predict the future power load demand more accurately and improve the accuracy and stability of the prediction. The model integrating CNN and BiLSTM can better capture the spatial and temporal characteristics of data in power load demand forecasting and improve the accuracy of forecasting. The features are extracted by CNN model and passed to BiLSTM model for sequence modeling. The model can better understand the complexity of power load data and make more reliable predictions. In a word, by integrating the advantages of CNN and BiLSTM, CNN-BiLSTM model can process time series data more effectively, and improve the accuracy and stability of forecasting, thus playing an important role in energy industry.

When using CNN network to extract the features of the power data in the original power system, preprocess it first. If the original power data is as shown in Eq. (1):

1 $$\Phi = \left\{ {\varphi_{1} ,\varphi_{2} , \ldots ,\varphi_{N} } \right\}.$$

Let *T* be the length of time series, that is, predict the ith point and take the previous *T* points as input, then the data set $\Psi$ can be expressed as Eq. (2):

2 $$\Psi = \left\{ {\Psi_{1} ,\Psi_{2} , \ldots ,\Psi_{n - T} } \right\}.$$

$\Psi_{i}$ can be expressed as Eq. (3):

3 $$\Psi_{i} = \left\{ {\varphi_{j - L} ,\varphi_{j - L + 1} , \ldots ,\varphi_{j - 1} } \right\},\;1 \le i \le n - L,\;j = i + L,i,j \in N.$$

Equation (4) serves as the conversion function, and the data is processed using the min–max standardization method to increase the model algorithm’s operational efficiency.

4 $$x_{j}^{\prime} = \frac{{x_{i} - x_{\min } }}{{x_{\max } - x_{\min } }}.$$

$x_{i}$ refers to raw data and $x_{j}{\prime}$ refers to standardized data.

A dropout layer is added between CNN feature extraction block and BiLSTM sequence prediction to prevent over-fitting. Then, BiLSTM network is used to effectively process the input characteristic information sequence data, which can capture the dependence between data information in power system.

The features $x_{t}$ and $h_{t - 1}$ of data information in the power system are input into the BiLSTM model, and the input data are obtained by sigmoid function, and the coefficients $f_{t} ,i_{t}$ are input by activation function, and the temporary unit variable $\tilde{c}_{t}$ is obtained. The calculation process is shown in Eqs. (5)–(7):

5 $$f_{t} = \delta \left( {\left[ {W_{f} \cdot \left[ {h_{t - 1} ,x_{t} } \right] + b_{f} ,W_{f}^{\prime} \cdot \left[ {h_{t - 1}^{\prime} ,x_{t} } \right] + b_{f}^{\prime} } \right]} \right),$$

6 $$i_{t} = \delta \left( {\left[ {W_{i} \cdot \left[ {h_{t - 1} ,x_{t} } \right] + b_{i} ,W_{i}^{\prime} \cdot \left[ {h_{t - 1}^{\prime} ,x_{t} } \right] + b_{i}^{\prime} } \right]} \right),$$

7 $$\tilde{C}_{t} = \tanh \left( {\left[ {W_{C} \cdot \left[ {h_{t - 1} ,x_{t} } \right] + b_{c} ,W_{c}^{\prime} \cdot \left[ {h_{t - 1}^{\prime} ,x_{t} } \right] + b_{c}^{\prime} } \right]} \right).$$

$\sigma$ and tanh are activation functions, and the former is sigmoid function. $W_{f} ,W_{i} ,W_{C} ,W_{o}$ are weight parameters. $h_{t - 1}$ refers to the output of the previous neuron. $C_{t}$ refers to the cell state at time* t*. $h_{t}$ refers to the hidden layer output at time t, and b refers to the bias vector. Then, the hidden state h_t_ at t can be expressed as Eqs. (8)–(10):

8 $$\vec{h}_{t} = \overrightarrow {LSTM} \left( {\vec{W}_{t} ,\vec{h}_{t - 1} ,\vec{b}_{t} ,\vec{c}_{t - 1} } \right),$$

9 $$\mathop{h}\limits^{\leftarrow} _{t} = \overleftarrow {LSTM} \left( {\mathop{W}\limits^{\leftarrow} _{t} ,\mathop{h}\limits^{\leftarrow} _{t - 1} ,\mathop{b}\limits^{\leftarrow} _{t} ,\mathop{c}\limits^{\leftarrow} _{t + 1} } \right),$$

10 $$h_{t} = \left[ {\vec{h}_{t} ,\mathop{h}\limits^{\leftarrow} _{t} } \right].$$

*W* and *b* respectively represent the relative weights of the door unit and the memory cell. *c*_*t*_ and* h*_*t*_ respectively represent the state of the memory cell and the hidden state of LSTM at *t*. → and ← represent forward data feature prediction and reverse data feature prediction respectively.

In the process of back propagation, the parameters in the model should be updated in the direction of the fastest gradient decline. Assume that the network parameter is $\theta$, the learning rate is $\eta$. The function represented by the network is $J\left( \theta \right)$. The maximum gradient of the function to $\theta$ at this time can be expressed as $\nabla_{\theta } J\left( \theta \right)$, so the updating equation of parameters can be expressed as Eq. (11):

11 $$\theta = \theta - \eta \nabla_{\theta } J\left( \theta \right).$$

In order to further optimize the problem that the loss function has too large swing amplitude in the update of the model and accelerate the convergence speed of the function, RMSProp algorithm uses the differential square weighted average for the gradient of weight *W* and offset* b*, as shown in Eqs. (12)–(15):

12 $$s_{dW} = \beta s_{dW} + \left( {1 - \beta } \right)dW^{2} ,$$

13 $$s_{db} = \beta s_{db} + \left( {1 - \beta } \right)db^{2} ,$$

14 $$W = W - \alpha \frac{dW}{{\sqrt {s_{dW} } + \varepsilon }},$$

15 $$b = b - \alpha \frac{db}{{\sqrt {s_{db} } + \varepsilon }}.$$

$s_{dW}$ and $s_{db}$ refer to the weighted average of exponential squares initialized to zero, respectively, and $\beta$ refers to momentum.

**Figure 2:**
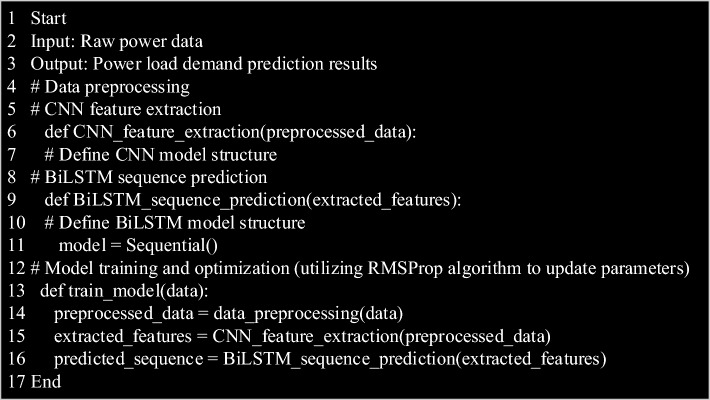
Pseudo-code flow charts of CNN-BiLSTM algorithm applied to power load demand forecasting.

In this model, the pseudo code of CNN-BiLSTM algorithm applied to power load demand forecasting is shown in Fig. 2.

In Fig. 2, firstly, CNN-BiLSTM algorithm receives raw power data as input, and outputs the prediction result of power load demand. In the data preprocessing stage, the original data is preprocessed to prepare for the subsequent feature extraction. Then, CNN model is used to extract features from the preprocessed data, and a CNN feature extraction function is defined to ensure that the model can effectively extract important spatial features from the data. Then, BiLSTM model is used to predict the sequence of features extracted from CNN to capture the time dynamic features in the data, and this process is realized by the defined BiLSTM sequence prediction function. Finally, RMSProp algorithm is used to train and optimize the model to improve the performance and prediction accuracy of the model. After the whole process, the algorithm gives the prediction results of future power load demand. Through this algorithm flow, CNN-BiLSTM model can be effectively applied to accurately predict the power load demand, which provides important support and guidance for the management and operation of the energy industry.

### Application of DBN in power system fault prediction and analysis

**Figure 3:**
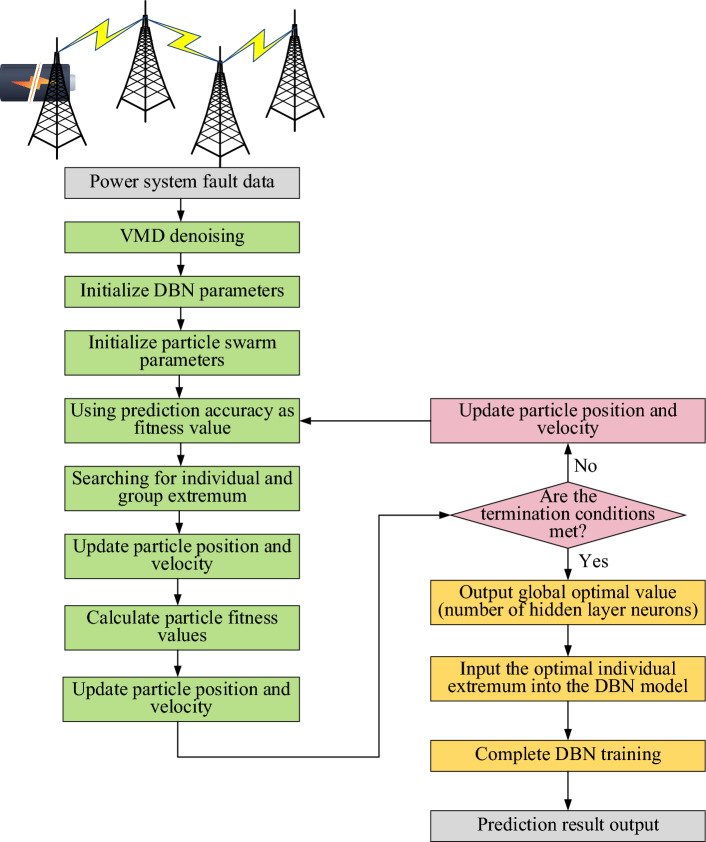
Power grid fault diagnosis model based on PSO algorithm and DBN.

At present, with the concept of smart grid put forward, the establishment of intelligent information operation and maintenance platform store a lot of data, and the large-scale access of distributed power sources increases the nonlinearity and uncertainty of data, which makes the fault analysis of power system more difficult. Aiming at the fault analysis of power system under the new situation, this study proposes a power grid fault diagnosis model based on PSO algorithm and DBN, as shown in Fig. 3.

In Fig. 3, firstly, power system fault data is input, and the input data is denoised by Variational Mode Decomposition (VMD)^25^. Then, the parameters of DBN^26^ and PSO algorithm^27^ are initialized. The model takes the accuracy of fault prediction as the fitness function of PSO algorithm to guide it to search for individual and global optimal solutions. PSO algorithm constantly updates the position and velocity of particles to converge towards the optimal solution step by step. If the requirements are satisfied, the global optimal value—which denotes the ideal number of neurons in the hidden layer—is produced by iterative evaluation. In the event that the termination condition is not satisfied, the PSO algorithm will go on finding the optimal architecture while updating the particle’s position and speed. The optimal individual value, or the ideal number of neurons in the hidden layer, is sent into the DBN model for training once the termination condition is satisfied. Finally, DBN training is completed and the identification results of power grid faults is output.

In this model, the fault parameter estimation is defined as Eq. (16):

16 $$\hat{\beta }_{k - 1,k}^{i} = g\left( {\overline{y}_{k} ,W_{k}^{i} ,V_{k}^{i} } \right).$$

$\hat{\beta }_{k - 1,k}^{i}$ is the *i*-th fault parameter when calculating *k*-1 at time *k*. $W_{k}^{i} ,V_{k}^{i}$ are the weight matrix of the output layer and hidden layer of the i-th DBN. $\overline{y}_{k}$ is the input vector of the DBN, and *g* is the nonlinear mapping realized by the DBN. During training, the Lyapunov function^28^ changes as shown in Eq. (17):

17 $$\Delta L_{k} = \frac{1}{2}\left( {e_{k + 1}^{2} - e_{k}^{2} } \right).$$

$e_{k}^{{}}$ refers to learning error. Let $G_{k} = \partial J_{k} /\partial W_{k} ,G_{k\max } = \mathop {\max }\limits_{k} ||G_{k} ||$. Because of $\eta_{1} = \eta_{w} G_{k\max }^{2}$, the Eq. (18) is defined:

18 $$\lambda = \frac{1}{2}||G_{k} ||^{2} \eta_{1} \left( {2 - \eta_{1} ||G_{k} ||^{2} } \right).$$

If the condition satisfying the convergence of the DBN is $\Delta L_{k} < 0$, then $\lambda > 0$. The following equation can be obtained from Eq. (18).

19 $$0 < \eta_{1} < \frac{2}{{||G_{k} ||^{2} }}.$$

If Eq. (19) is satisfied in the DBN, the network model can be kept stable and convergent, thus the state and output of the power system can be predicted.

The fault diagnosis and prediction framework can be divided into the following steps:

1. Data preprocessing. Input power system fault data, which may include power grid operation status, equipment sensor data, etc. Variational Modal Decomposition (VMD) is used to denoise the data, so as to reduce the interference noise in the data and improve the robustness and accuracy of the subsequent model.
2. Model initialization. Particle swarm optimization (PSO) is used to initialize the parameters of deep belief network (DBN) and PSO. DBN is a neural network model with multiple hidden layers, which is used for fault diagnosis and prediction.
3. PSO process. PSO algorithm takes the accuracy of fault prediction as the fitness function, and constantly updates the position and speed of particles to find the optimal parameter configuration of neural network. Through iterative optimization, PSO algorithm guides particles to search in the direction of global optimal solution.
4. PSO algorithm termination conditions. PSO algorithm will continue to iterate and update until the termination conditions are met, such as the maximum number of iterations, accuracy requirements or other preset conditions.
5. Once the PSO algorithm meets the termination conditions, it outputs the global optimal value, which represents the optimal number of neurons in the hidden layer. This value will be used as a key parameter for subsequent DBN model training.
6. DBN model training. The optimal number of hidden layer neurons determined by PSO algorithm will be used as the hidden layer configuration of DBN model, and then the DBN model will be trained. This step may involve supervised learning with fault data, so that the model can accurately identify power grid faults.
7. Output of fault diagnosis results. After completing the training of DBN model, the model will be used to identify and predict power grid faults. Finally, through this process, the identification results of power grid faults are output, which provides guidance for subsequent fault treatment and maintenance.

This framework integrates data preprocessing, PSO and DBN to effectively diagnose and predict power grid faults and improve the accuracy and robustness of power system faults.

### Experimental analysis

In order to analyze the performance of the power load demand forecasting model based on CNN-BiLSTM, the smart meter data of XX community in Xi’an from October 2021 to October 2022 are collected. The original power load data is collected every 6 s by the home smart meter, but this study analyses and forecasts the 5-min power load data and needs to convert the data unit. The load data is converted into electricity consumption within 5 min, and then the data are integrated, and 36,105 power data are obtained after integration. According to a 7:3 ratio, power statistics are split at random into training and test sets. The model makes use of a number of Python modules and the TensorFlow simulation framework. There are 100 iterations and a batch size of 100 in the particular super parameter settings. The random gradient descent algorithm is utilized to optimize the loss function, with a starting learning rate of 0.001. The model techniques presented by CNN, BiLSTM, LSTM^29^, and Ahmadian et al. are utilized to evaluate the model’s performance in this study using three different metrics: root mean square error (RMSE), mean absolute error (MAE), and mean absolute percentage error (MAPE).

Furthermore, the performance of the power grid fault diagnosis model based on PSO algorithm and DBN is evaluated. The simulation experiment is completed on an Inter Core i7-9750H personal computer with a single CPU of 2.6 GHz, 16 GB of memory and 64-bit operating system. Because the accuracy of model identification will change with the number of hidden layers, DBN models with hidden layers of 1, 2, 3, 4 and 5 are built respectively. The number of neurons in each layer is set to 200, the number of iterations is set to 100, the loss function is cross entropy, the learning rate adopts random gradient descent algorithm, and the initial learning rate is set to 0.001. The improved 10 kV distribution network model of IEEE13 nodes is established, the arc fault module is analyzed, and the early fault model is completed. Half-cycle early fault, multi-cycle early fault, fixed impedance grounding, motor start-up and load switching are respectively set, and the load of each fault branch is respectively set to light load, heavy load, and full load. When the motor is started, three groups of situations with different power levels are set according to the power level of the motor. The switching time of capacitors is set to 9 random values in a cycle, and 12 groups of capacitors with different sizes are set. According to the above simulation conditions, the obtained data are parameterized, and a total of 8517 groups of samples are obtained. The training set consists of 70% data samples of various state types, and the remaining 30% samples constitute the test set. Compared with the model algorithms proposed by DBN, General Regression Neural Network (GRNN)^30^ and Hong et al. in terms of accuracy and training time.

## Results

### Power load demand forecasting analysis with different model algorithms

**Figure 4:**
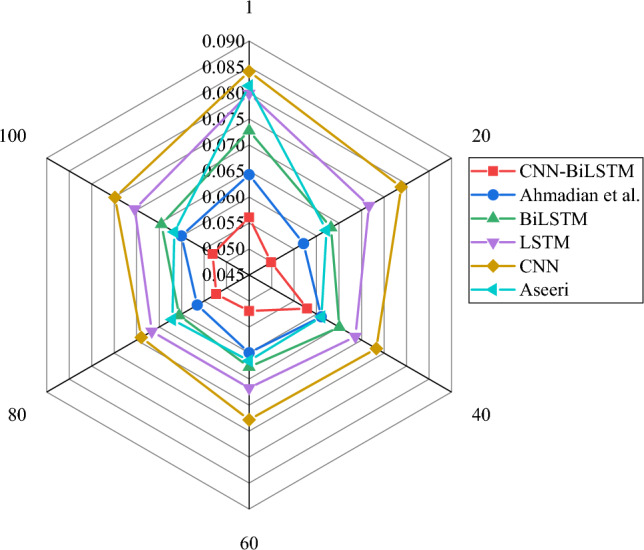
RMSE result of power load forecasting with different algorithms.

Figures 4 and 6 illustrate the evaluation of the CNN-BiLSTM power load demand forecasting model algorithm used in this study and the model algorithms proposed by CNN, BiLSTM, LSTM, Ahmadian et al. and Aseeri^31^ from the perspectives of RMSE, MAE, and MAPE, respectively. Meanwhile, the average values of each algorithm under RMSE, MAE, and MAPE indicators are shown in Table 2.

**Table 2:** The average results of each algorithm under RMSE, MAE, and MAPE indicators.

Indicators	CNN-BiLSTM	Ahmadian et al.	BiLSTM	LSTM	CNN	Aseeri
RMSE	0.0535	0.0598	0.0648	0.0707	0.0755	0.0617
MAE	0.0764	0.0845	0.0916	0.0975	0.1052	0.0891
MAPE	0.1023	0.1133	0.1229	0.1365	0.1494	0.1193

**Figure 5:**
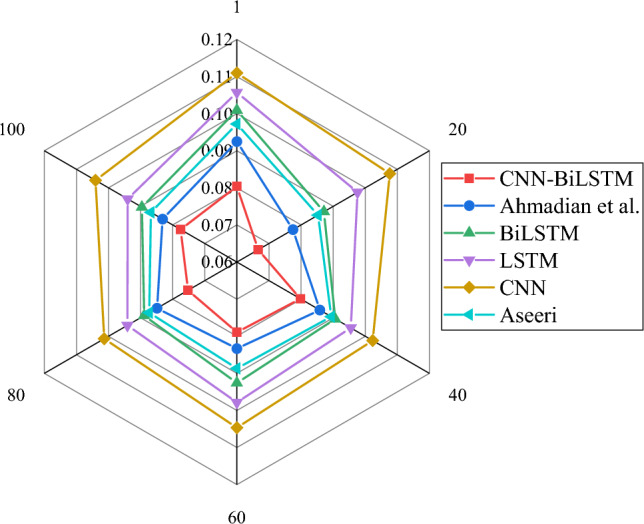
MAE result of power load forecasting with different algorithms.

**Figure 6:**
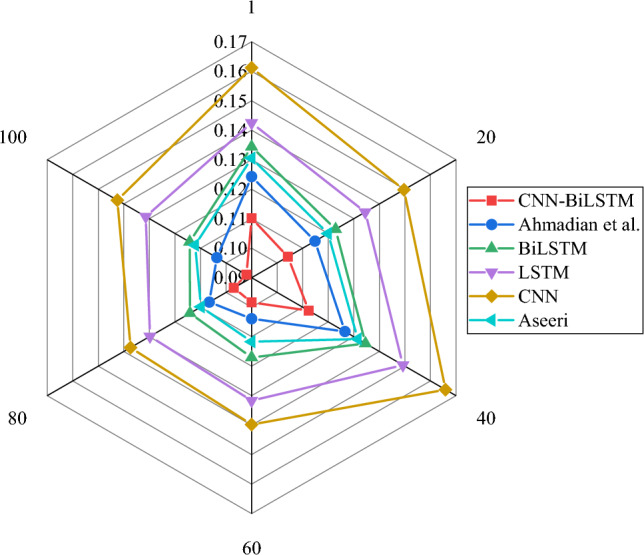
MAPE result of power load forecasting with different algorithms.

In Figs. 4, 5 and 6 and Table 2, the results that the errors of RMSE, MAE and MAPE change with the increase of iteration times. By comparing the error change results of each algorithm, it shows that the average RMSE, MAE and MAPE of CNN-BiLSTM algorithm proposed in this study are 0.054, 0.076 and 0.102, respectively, while the error values of other model algorithms are obviously higher than those of the algorithm model proposed in this study. Additionally, the model algorithms for this study < model algorithm suggested by Ahmadian et al.^20^ < Aseeri^31^ < BiLSTM < LSTM < CNN, and the recognition errors RMSE, MAE, and MAPE of each algorithm are the model algorithms. As a result, the CNN-BiLSTM-based power load demand forecasting model in this study has higher power load forecasting accuracy when compared to other researchers’ algorithms. This means that the model can more accurately support the low-carbon intelligent development of the power system in the energy industry by better predicting the load demand in the power system.

### Prediction and analysis of power grid fault diagnosis with different model algorithms

**Figure 7:**
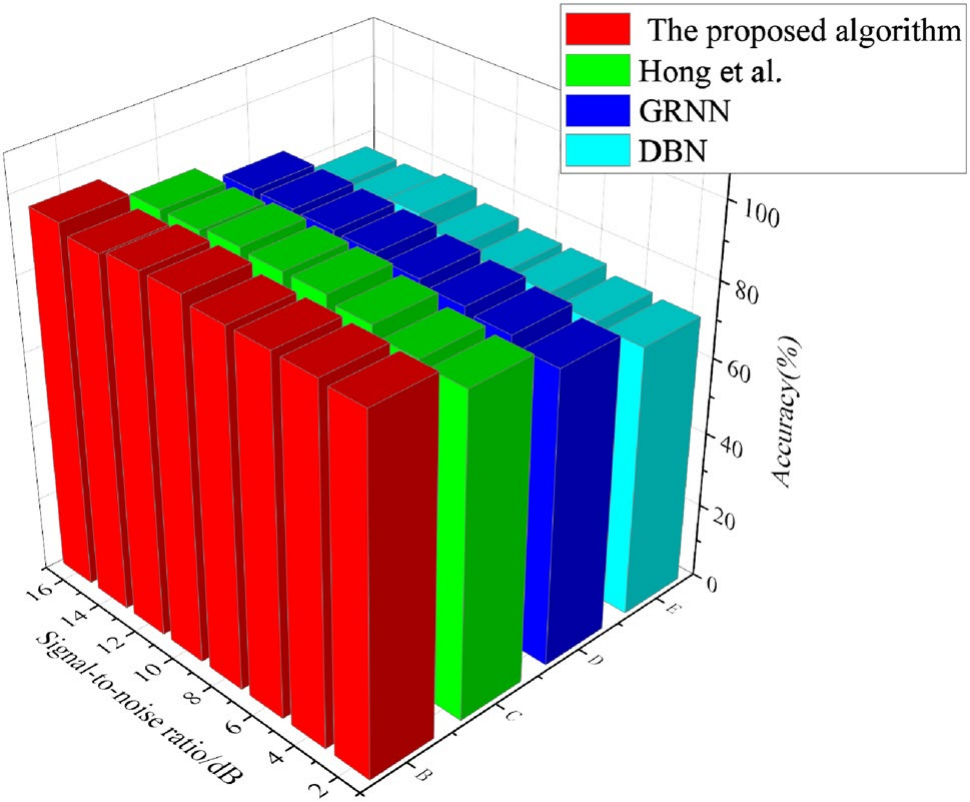
Result of fault prediction accuracy of different algorithms.

**Figure 8:**
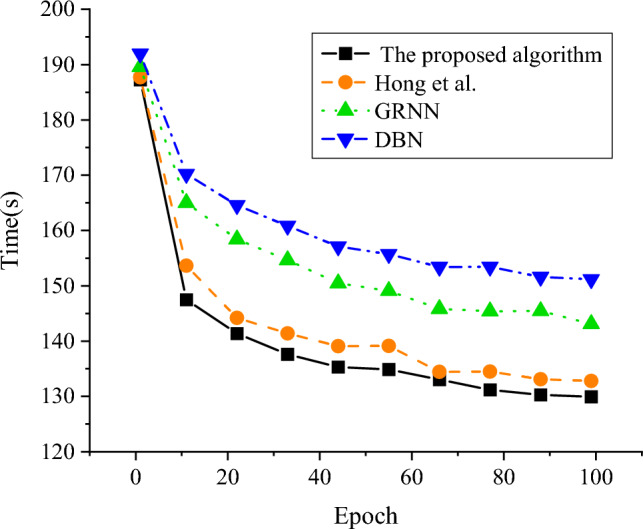
Result of time required for fault prediction of different algorithms.

The power grid fault diagnosis model constructed in this study is compared with the model algorithm proposed by DBN, GRNN and Hong et al.^18^, and the results of accuracy and training time are shown in Figs. 7 and 8.

In Fig. 7, comparing the fault prediction model algorithm constructed in this study with the model algorithm proposed by DBN, GRNN and Hong et al.^18^ it shows that the fault prediction accuracy of the model algorithm proposed in this study reaches 96.22%, which is 2.30% higher than that of the model algorithm proposed by Hong et al.^18^. Meanwhile, the order of prediction accuracy of each algorithm is the proposed algorithm > Hong et al.^18^ > GRNN > DBN. In Fig. 8, it shows that with the increase of iteration times, the prediction time required by this model algorithm is obviously lower than other algorithms, reaching 129.94 s. It indicates that compared with other scholars’ algorithms, the power grid fault diagnosis model based on PSO algorithm and DBN constructed in this study has higher power grid fault prediction accuracy, higher fault prediction efficiency, better noise resistance and stability.

## Discussion

Through the analysis of the above results, it shows that the CNN-BiLSTM and PSO-DBN models proposed in this paper have achieved remarkable advantages in power load demand forecasting and power grid fault diagnosis. Firstly, from the aspect of load demand forecasting, the average RMSE, MAE and MAPE of CNN-BiLSTM model are 0.0535, 0.0764 and 0.1023, respectively, which are significantly lower than other model algorithms. Secondly, by comparing the forecasting accuracy of different models, it is found that the performance of this model in power load demand forecasting is better than other researchers’ algorithms, which is CNN-BiLSTM model > Ahmadian et al.^20^ model > BiLSTM > LSTM > CNN. Echoing the views of Shi et al.^32^ and Anu Shalini and Sri Revathi^33^, this means that the model in this paper can more accurately predict the load demand of power system and help support the energy industry to develop into low-carbon intelligence. However, its scope of application may be affected by factors such as power system structure, energy types and seasonal changes. If the model is verified on data sets that fully represent different geographical and climatic conditions, the adaptability of the model to different power system scenarios can be better understood.

For power grid fault diagnosis, the proposed model is compared with other algorithms (such as the model proposed by DBN, GRNN and Hong et al.^18^). The results show that the fault prediction accuracy of this model reaches 96.22%, which is 2.30% higher than the model proposed by Hong et al.^18^. In addition, the ranking of fault prediction accuracy is the proposed model, Hong et al.^18^ model, GRNN, and DBN. In terms of prediction time, the time required by this model is obviously lower than other algorithms, only 129.94 s. This is consistent with the conclusion of Sun et al.^34^, which shows that PSO-DBN model is superior in accuracy and efficiency compared with other scholars’ algorithms in power grid fault diagnosis. However, factors such as power system structure, equipment type and operating environment may affect the applicability of the model. In order to evaluate the universality of the model, it is necessary to verify the data of different types of power grids, power systems of different scales and different regions. This ensures that the model can perform well in various power system scenarios.

Therefore, the CNN-BiLSTM and PSO-DBN models are excellent in load demand forecasting and power grid fault diagnosis, and have higher forecasting accuracy and efficiency than other algorithms. These results are of great significance for promoting the sustainable development of energy industry and the intelligent development of power system.

## Conclusions

Firstly, a CNN-BiLSTM-based power load demand forecasting model is built in this study. Through the analysis of its RMSE, MAE and MAPE error results, it reaches 0.054, 0.076 and 0.102 respectively, which is obviously better than the identification error of the model algorithm proposed by Ahmadian et al.^20^, showing higher forecasting accuracy. In the aspect of fault prediction, the power grid fault diagnosis model algorithm based on PSO algorithm and DBN proposed in this study shows 96.22% prediction accuracy, which is obviously improved compared with other models, such as DBN, GRNN and previous research models. This shows the superiority of this model in power load demand forecasting, and provides more accurate support for the low-carbon intelligent development of power system.

However, this paper also has some limitations, such as data availability and quality, data acquisition difficulties and data quality inconsistency, which may affect the training and accuracy of the model. In addition, the integration of the model into a complex power system may be limited by technology, and the limitations of system integration and actual operation need to be considered. Therefore, in the follow-up research, people can explore multi-source data integration and data cleaning technology, and focus on customizing the model to adapt to different scenarios. In addition, the optimization of system integration and the operational practicability of the model are also key, so people need to pay attention to the practical feasibility study and algorithm efficiency optimization. To sum up, solving the problems of data availability, system integration, and operation limitation will be the key direction of future research to improve the feasibility and effectiveness of CNN-BiLSTM and PSO-DBN models in practical application of power system.

## Data Availability

The data presented in this study are available on request from the corresponding author.
